# Development of novel methods that monitor necroptosis and the release of DAMPs at the single cell resolution

**DOI:** 10.15698/cst2019.02.177

**Published:** 2019-01-22

**Authors:** Hiroyasu Nakano, Shin Murai, Yoshifumi Yamaguchi, Yoshitaka Shirasaki, Osamu Nakabayashi, Soh Yamazaki

**Affiliations:** 1 Department of Biochemistry, Toho University School of Medicine, 5-21-16 Omori-Nishi, Ota-ku, Tokyo 143-8540, Japan.; 2 Host Defense Research Center, Toho University School of Medicine, 5-21-16 Omori-Nishi, Ota-ku, Tokyo 143-8540, Japan.; 3 Hibernation metabolism, physiology, and development Group, Environmental Biology Division, Institute of Low Temperature Science, Hokkaido University, Kita 19, Nishi 8, Kita-ku, Sapporo, Hokkaido 060-0819, Japan.; 4 Precursory Research for Embryonic Science and Technology, Japan Science and Technology Agency, Chiyoda-ku, Tokyo 102-0075, Japan.; 5 Department of Biological Sciences, Graduate School of Science, The University of Tokyo, 7-3-1 Bunkyo-ku, Tokyo 113-0033, Japan.

**Keywords:** danger-associated molecular pattern (DAMP)s, the endosomal sorting complex required for transport (ESCRT) complex, Forster resonance energy transfer (FRET), high mobility group (HMGB)1, mixed lineage kinase domain-like protein (MLKL), necroptosis, receptor-interacting kinase (RIPK)3, total internal reflection fluorescent microscope (TIRF-M)

## Abstract

Necroptosis is a regulated form of necrosis that depends on receptor-interacting protein kinase (RIPK)3 and mixed lineage kinase domain-like protein (MLKL). While danger-associated molecular pattern (DAMP)s are released from dead cells and involved in various pathological conditions, the mechanisms underlying regulation of the release of DAMPs are not fully understood. Apoptosis and pyroptosis can be detected by several types of sensors such as Forster resonance energy transfer (FRET) biosensors, termed SCAT1 (a sensor for caspase 1 activation based on FRET) and SCAT3, respectively. These sensors have provided better understanding of pyroptosis and apoptosis *in vitro* and *in vivo*. However, there have been no biosensors to monitor necroptosis. Development of a FRET biosensor that monitors necroptosis and generation of transgenic mice expressing such FRET biosensor might be useful to understand the mechanisms underlying the execution of necroptosis and also the consequences of necroptosis *in vivo*. In our recent study (Nat Commun, 9(1):4457), we developed a FRET biosensor for necroptosis, termed SMART (a sensor for MLKL activation by RIPK3 based on FRET). SMART is composed of a fragment of MLKL and monitors necroptosis, but not apoptosis or necrosis. Moreover, we recently developed a platform called Live-Cell Imaging for Secretion activity (LCI-S) to monitor protein secretion at the single cell level. This platform has enabled us to monitor the release of HMGB1 (High Mobility Group Box 1), one of the DAMPs, at the single cell level and reveals two different modes of the release of HMGB1 from necroptotic cells.

## HOW DOES SMART SENSE THE EXECUTION OF NECROPTOSIS?

**Figure 1:**
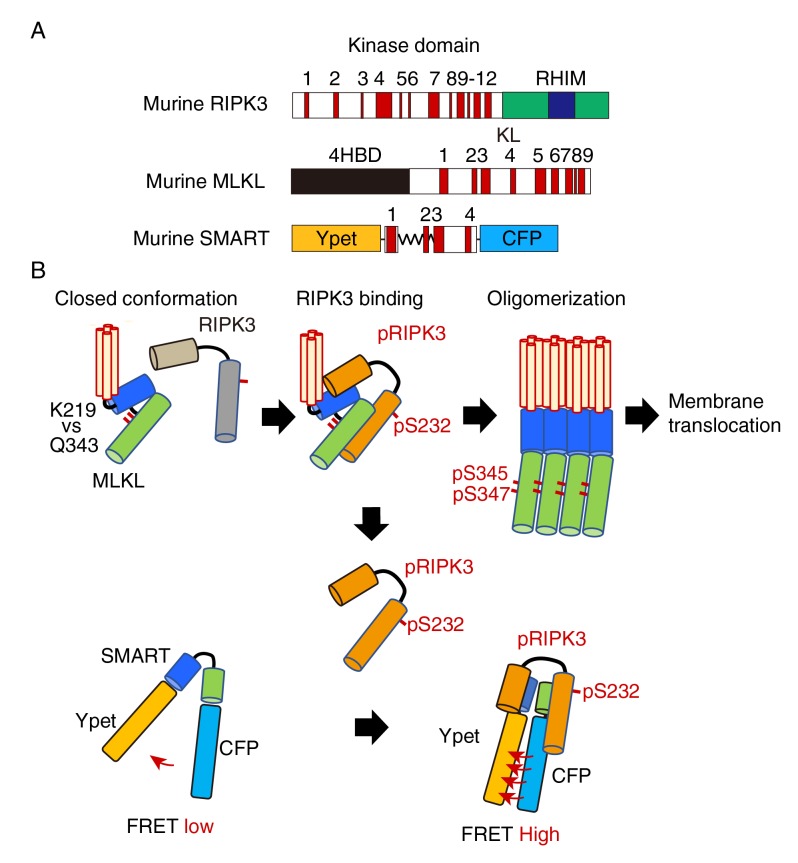
FIGURE 1: A model for the mechanism how SMART senses necroptosis. **(A)** Domain structures of murine RIPK3, MLKL, and diagram of SMART. mRIPK3 is composed of N-terminal kinase domain that contains twelve helices and C-terminal RIP homotypic interaction motif (RHIM) domain. mMLKL is composed of N-terminal four helices bundle domain (4HBD) and C-terminal kinase-like (KL) domain. mSMART is composed of N-terminal Ypet and C-terminal CFP where a modified fragment containing α 1 to 4 helices of MLKL is inserted in the intervening region. Numbers indicate each α helix. (B) In unstimulated cells, endogenous MLKL adopts an inactive conformation via hydrogen bond between K219 and Q343, thereby suppressing spontaneous oligomer formation. Following necroptosis induction, activated RIPK3 (pRIPK3) binds to MLKL, resulting in phosphorylation of MLKL that induces conformational changes and subsequent oligomer formation. Oligomerized MLKL translocates to the plasma membrane and induces membrane rupture. Since SMART does not contain K216, SMART appears to adopt an open conformation. Weak FRET signals of SMART are detected in unstimulated cells. Once the oligomerized MLKL is released from activated RIPK3 and translocates to the plasma membrane, activated RIPK3 subsequently binds to induces conformational changes of SMART, increasing the FRET/CFP ratio. Thus, an increase in the FRET/CFP ratio of SMART is tightly correlated with plasma membrane translocation of oligomerized MLKL.

Various types of cells undergo necroptosis following stimulation with death ligands including tumor necrosis factor, Fas ligand, and TRAIL, or polyI:C, and viral infection. Among various molecules involved in regulation of necroptosis, RIPK3 and MLKL are essential key players (**Figure 1A**). Upon stimulation with death ligands, activated RIPK3 interacts with and phosphorylates MLKL, resulting in oligomerization and plasma membrane translocation of MLKL (**Figure 1B**). To develop a FRET biosensor that monitors necroptosis, we focused on conformation changes of MLKL induced by RIPK3 binding. We generated various FRET biosensors containing a series of fragments of MLKL between modified yellow fluorescent protein (Ypet) and enhanced cyan fluorescent protein (ECFP). We finally developed a FRET bio-sensor, termed SMART (**Figure 1A**). SMART enabled us to detect necroptosis by monitoring an increase in the FRET/CFP ratio in murine cells including L929 cells, embryonic fibroblast (MEF)s, dermal fibroblasts, and a colon epithelial cell line, aMoC1 cells. Since murine SMART did not work well in human cells, we also generated a human version of SMART.

We next investigate the mechanisms underlying an increase in the FRET/CFP ratio of SMART. In unstimulated cells, unphosphorylated MLKL forms a closed conformation via hydrogen bond between lysine at 219 (K219) and glutamine at 343 (Q343), thereby preventing spontaneous oligomerization (**Figure 1B**). Phosphorylation of MLKL by RIPK3 disrupts the hydrogen bond between K219 and Q343, resulting in its oligomerization and membrane translocation that subsequently induces membrane permeabilization. siRNA-mediated knockdown of *Ripk3* or *Mlkl*, or using *Mlkl*-deficient MEFs revealed that endogenous RIPK3 and MLKL are required for an increase in the FRET/CFP ratio of SMART. Unexpectedly, mutation of phosphorylation sites of SMART by RIPK3 per se did not affect the ability to sense necroptosis. Moreover, inducible expression of a constitutively active mutant of MLKL Q343A induced an increase in the FRET/CFP ratio of SMART, suggesting that SMART somehow senses the oligomerized MLKL. In contrast to murine MLKL, human MLKL contains a cysteine at 86 (C86) at the N-terminus that is a target of a necroptosis inhibitor, Necrosufonamide (NSA). NSA inhibits translocation of MLKL, but not oligomerization of MLKL. Taken that NSA also inhibited an increase in the FRET/CFP ratio of SMART, SMART might sense membrane translocation of oligomerized MLKL, but not the oligomerized MLKL itself. How do we integrate these findings and make a model for the mechanism underlying an increase in the FRET/CFP ratio of SMART? Notably, the interaction of SMART with RIPK3 was required for an increase in the FRET/CFP ratio of SMART and the majority of cytoplasmic MLKL translocated into the plasma membrane after necroptosis induction. Thus, once the oligomerized MLKL is released from activated RIPK3 and translocates to the plasma membrane, activated RIPK3 subsequently binds to induce conformational changes of SMART, increasing the FRET/CFP ratio (**Figure 1B**). However, this model appears to be inconsistent with the results that an increase in the FRET/CFP ratio of SMART was abolished in *Mlkl*-/- MEFs. Further studies will be required to refine this model.

## REAL TIME MONITORING OF THE RELEASE OF DAMPs FROM A SINGLE CELL

**Figure 2:**
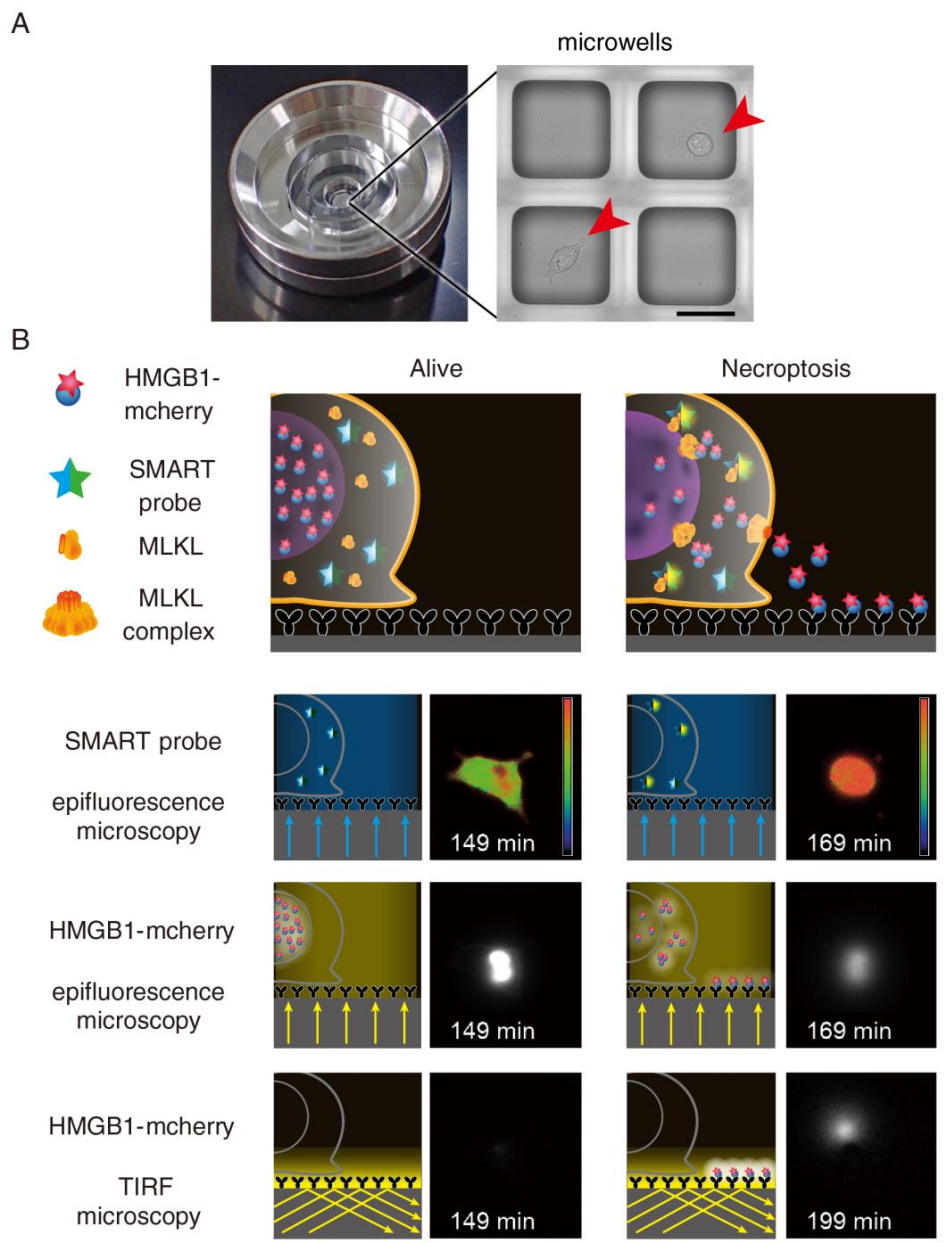
FIGURE 2: Detection of the release of HMGB1-mCherry using TIRF microscopy. **(A)** A single chamber well is composed of approximately 1,000 sub-nanolitre-sized microwells with 80 μm square in size where a single cell is seeded onto one microwell. Scale bars indicate 50 μm. Arrowheads indicate individual cell. **(B)** LCI-S monitors an increase in the FRET/CFP ratio of SMART, HMGB1-mCherry signals in the nucleus by epifluorescence microscopy, and released HMGB1-mCherry by TIRF-M. Microwells were precoated with anti-RFP antibody, and released HMGB1-mCherry was rapidly captured by anti-RFP antibody and was detected by TIRF-M. L929 cells were untreated (Alive) or treated with TNF and zVAD-fmk to induce necroptosis (Necroptosis). Fluorescent signals were analyzed at every 2 min and representative images of live and necroptotic cells are shown.

We previously developed the real-time single cell secretion assay platform termed Live cell imaging for secretion activity (LCI-S) that monitors cytokine secretion at the single-cell resolution. This system is composed of a cell culture chamber containing approximately 1,000 sub-nanolitresized microwells with 80 μm square in size (**Figure 2a**). Hundreds of cells are randomly seeded onto the microwell array. Among them, microwells contain a single cell are selected and analyzed. The released cytokines from a single cell are rapidly captured by a precoated antibody and then visualized with a fluorescence dye-conjugated detection antibody using total internal reflection fluorescent microscopy (TIRF-M). This platform has enabled us to monitor secretion of cytokines such as interleukin (IL-1)β or IL-6 at the single-cell resolution. Although we tried to apply LSI-S to monitor HMGB1 release from necroptotic cells, commercially available antibodies against HMGB1 were not suitable for LCI-S. Thus, we generated cells stably expressing HMGB1 fused to mCherry and then microwells were coated with anti-Red fluorescent protein (RFP) antibody (**Figure 2b**). Upon necroptosis induction, HMGB1-mCherry was released from necroptotic cells and the released HMGB1-mCherry was efficiently and rapidly visualized by TIRF-M. Notably, LCI-S can monitor any DAMPs when cells express a DAMP as a fusion protein with a fluorescence protein such as mCherry or GFP.

## MODIFIED LCI-S REVEALS TWO DIFFERENT MODES OF THE RELEASE OF HMBG1

There are many DAMPs released from dying cells, including HMGB1, histone H3, and heat shock proteins. LCI-S revealed that HMGB1-mCherry, but not histone H3-mCherry was released from L929 cells undergoing necroptosis, suggesting that the release of HMGB1 and histone H3 is regulated in their nature or cell-type specific manner. Moreover, the translocation of HMGB1-mCherry from the nucleus to the cytosol preceded before the plasma membrane rupture. This indicates that MLKL-dependent permeabilization of the nuclear membrane starts earlier than that of the plasma membrane. Unexpectedly, we found that the duration of the release of HMGB1 was divided into two modes; a burst-mode and a sustained-mode. In a sustained mode cell, the release of HMGB1 continued more than 100 min, whereas the release of HMBG1 terminated within 10 min in a burst-mode cell. At this moment, the biological significance of two different modes of the release of HMGB1is unclear, one might surmise that a burst-mode cell might elicit strong immune responses to the surrounding microenvironment. Alternatively, sustained release of HMGB1 might induce another cellular response, such as recruitment of immune cells.

Previous studies have shown that the endosomal sorting complex required for transport (ESCRT)-III complex is involved in membrane repair, virus budding, and multivesicular body formation. Recent studies have reported that MLKL-containing vesicles are released from dying cells. Taken that knockdown of components of the ESCRT-III complex such as *Chmp4b* or *Chmp2a* exacerbates TNF-induced necroptosis, the release of MLKL-containing vesicles appears to counteract cell death induction. Intriguingly, knockdown of *Chmp4b* completely abolished cells showing a sustained mode release of HMGB1, and all *Chmp4b* knockdown cells exhibited a burst-mode release of HMGB1. Moreover, the expression of MLKL has been shown to be induced by virus infection or IFNα/β stimulation. Thus, the balance between MLKL and proteins associated with membrane repair such as CHMP4B might critically determine whether cells exhibit a sustained-mode or burst-mode of HMGB1 release.

